# Are Animal Models Needed to Discover, Develop and Test Pharmaceutical Drugs for Humans in the 21st Century?

**DOI:** 10.3390/ani10122455

**Published:** 2020-12-21

**Authors:** Pandora Pound

**Affiliations:** Safer Medicines Trust, P.O. Box 122, Kingsbridge TQ7 9AX, UK; pandora@safermedicines.org

Despite many decades of research, much of which has focused on studies in animals, we humans continue to suffer from multiple diseases for which there are no cures or treatments. It is now clear that insights provided by animal studies frequently fail to translate to humans, explaining the very high failure rate observed when new medicines are evaluated in clinical trials. Additionally, there is growing evidence that animal studies are often conducted and reported so poorly that no clear conclusions may be drawn from them. A range of initiatives is now underway to improve the quality of animal studies, in the hope that clinical benefits will eventually ensue. Running parallel to these initiatives, however, are calls to adopt novel, human biology-based approaches in drug discovery instead of animal models. It is argued that these new approaches (e.g., organoids, organs-on-a-chip) will revolutionise drug discovery and our understanding of human disease due to their greater relevance to humans and their superior predictive ability. So, which is the way forward? Should we continue to try to improve animal models or should we focus instead on exploring the potential of new approach methodologies (NAMs)?

Each of the six papers in this Special Issue addresses this question from a different perspective—no great surprise given that the authors hail from a variety of disciplines (including pharmaceutics, pharmacovigilance, research and innovation, population health, laboratory animal science, ethics, biological research, evidence-based health sciences and engineering) and a range of organisations (academia, industry, non-profit, executive body) and countries (Ireland, UK, Netherlands, Germany, Italy, Belgium, US). The papers themselves are diverse and include original research, systematic and narrative reviews and a conference report. Although the perspectives vary, all are agreed on one thing: there is a crisis in preclinical research that requires urgent attention.

Among those advocating improvements to animal models are Guilherme Ferreira and colleagues, who argue that efforts should be concentrated on improving the robustness of animal data [1]. Without good quality animal data, they suggest, it will not be possible to determine whether or not animal research is a valid paradigm for drug development. With this in mind, the authors invite us to consider three ways of improving external validity: first, by systematically identifying which aspects of a human disease are replicated in an animal model, to enable the selection of more predictive animal models; second, by combining animal and human data in systematic reviews and meta-analyses so that drug effect sizes in animals and humans can be compared and animal models ranked according to their predictive ability, and third; by comparing pharmacokinetic and pharmacodynamic data between preclinical studies and the corresponding clinical trials, to enhance the likelihood of selecting the most relevant animal model. Also highlighting the need for greater external validity are Cathalijn Leenaars and colleagues, who examine how well animal and human study designs align [2]. Their systematic review, conducted within the context of methotrexate for rheumatoid arthritis, found several differences between the animal and human study designs, including route of drug administration, measures taken to reduce the risk of bias and the sex and age of research subjects. The authors recommend that scientists performing animal and human studies adapt their designs to minimise misalignment and improve external validity, suggesting that this will eventually increase clinical translation.

Taking a different perspective, Francesca Pistollato and colleagues note that despite generous funding for research into Alzheimer’s disease (AD) and cancer over the last two decades—much of it involving animals—the failure rate in drug development remains 97% for cancer and 99% for AD, with patients and families continuing to suffer [3].They argue the case for monitoring the impact of biomedical research funding and note that the European Commission is currently taking steps to identify which scientific methods and research approaches underpin advances, potentially enabling the redistribution of resources if necessary. Lindsay Marshall and colleagues also observe that increased funding for cancer research has failed to translate into significant impacts for patients. They examine funding in relation to two specific types of cancer research: patient-derived xenografts (PDX), which involve injecting human tumour biopsies into animals, and patient-derived organoids (PDO), which use patient tumour samples to create in vitro models [4]. Focusing on breast, lung and colorectal cancers, the authors found that PDX is currently the preferred approach, receiving more funding and being associated with more publications and clinical trials than PDO models. However, they reveal a trend towards a gradual increase in funding for new PDO projects and the number of publications associated with PDO models, suggesting a shift towards human-relevant approaches within cancer research.

Human-relevant approaches are of interest to Dania Movia and Adriele Prina-Mello, who explore them in the context of orally inhaled drugs (OIDs) which are used to treat respiratory and other diseases [5]. Movia and Prina-Mello suggest that while the limitations of existing preclinical animal and in vitro models contribute to poor clinical translation in the field of OIDs, cell-based NAMs such as Air-Liquid Interface cell cultures, lung organoids and lungs-on-a-chip show exciting potential. Lungs-on-a-chip can be used across the board, they note, from basic research through toxicity testing to drug discovery. In the context of COVID-19, they suggest that lungs-on-a-chip enable modelling of the human response to lung infection by the influenza virus, or by viral pseudo particles expressing the spike protein of SARS-CoV-2, and allow the effects of existing and novel therapeutics to be explored. While not shying away from the limitations of NAMs, the authors argue that they are potentially more predictive of human outcomes and may save money by enabling compounds to fail earlier in the development process. They recommend that efforts are focused on validating NAMs for regulatory acceptance.

The final paper, by Merel Ritskes-Hoitinga and colleagues, is a report of an international conference held in the Netherlands in November 2019 [6]. The conference brought together speakers from epidemiology, sociology, animal research, ethics, industry and education to explore key questions about the scientific validity of preclinical animal models and to ask whether reliable translation from animals to humans will ever be possible. As the authors note, while some speakers believed this to be likely given sufficient effort, others felt that the low translational success of the animal model paradigm warranted serious discussion about its future. There was acknowledgement of an increasing emphasis on NAMs as well as a growing concern about the moral status of laboratory animals. In addition to recommending optimisation of the methodology and design of animal experiments, the authors advocate a greater role for patients in research (particularly in terms of determining the research agenda), collaboration between preclinical and clinical stakeholders, more focus on prevention and greater consideration of the potential of NAMs.

These six papers illustrate the existence of two separate movements within the scientific world at present—one to “improve animal research” and the other to “promote NAMs”. Each has its own particular challenges. The “improve animal research” movement faces two key problems. First, there is a lack of evidence that improvements in the quality of preclinical animal studies will actually improve clinical translation. For example, the STAIR recommendations, launched over twenty years ago to improve animal study design in the field of stroke, have failed to improve clinical translation [7]. In the same field, a neuroprotective drug developed according to the best preclinical and clinical guidelines failed to benefit humans despite being successful in animals [8]. Similarly, a recent hypothesis that animal toxicology studies, because they follow Good Laboratory Practice guidelines [9], would be more predictive when compared with efficacy studies and pharmacokinetic–pharmacodynamic studies using animals, was not confirmed [10]. The second major stumbling block is that even if animal studies were conducted and reported perfectly, species differences would still render the extrapolation of findings from animals to humans unreliable, even in the event that the most ‘appropriate’ animal model is selected. The consequences exerted by subtle differences between the species are an important but neglected aspect of external validity and have potential to significantly compromise clinical translation [11,12,13,14,15].

On the other hand, the “promote NAMs” movement is not without its problems. As Movia and Prina-Mello point out in this issue, many in vitro assays use transformed cell lines that may differ from their primary counterpart in terms of gene and protein expression [5]. Furthermore, in vitro studies are vulnerable to many of the same problems of experimental design as animal studies [16], and there is a need for greater standardization in cell culture (e.g., cell media, cell types, culture conditions, protocols used) in order to increase reproducibility [17]. Improved reporting is also necessary [18]. Other challenges include in vitro to in vivo extrapolation, obtaining data on ADME (absorption, distribution, metabolism and excretion) in the context of new compounds or administration routes [19], and data interpretation; many computational methods are somewhat “black box”, making it difficult to know which parameters and thresholds have been applied to reach the conclusions.

As noted above, the two movements have emerged in response to a crisis brought about by poor quality preclinical research and disappointing clinical translation rates. Failure to translate is clearly a significant anomaly for preclinical research. Writing in the 1960s, philosopher of science Thomas Kuhn observed that when confronted with anomalies within a paradigm, scientists tend not to renounce that paradigm but attempt to modify their theory in order to eliminate any conflict [20]. The “improve animal research” movement can be understood within this context, as an attempt to resolve the anomalies and make the paradigm work. As long as anomalies are regarded as superficial, argues Kuhn, there will be no revolution; an anomaly has to reveal real inadequacy in a paradigm to provoke radical change. The poor quality of animal research appears to be regarded as a relatively superficial issue that can be resolved, allowing the paradigm to continue. However, species differences present an altogether more challenging problem for the paradigm since these are insurmountable and will continue to make prediction from animals to humans unreliable [21]. The use of “humanised” (i.e., genetically modified) animals was an attempt to resolve this anomaly but failed to improve either the predictive ability of animal models or their clinical translation [22]. Despite this, the problem of species differences has not yet been widely recognised as a serious anomaly—possibly because all attention is currently focused on the research quality issue. It seems unlikely, however, that there will be any radical change in paradigm until the significance of species difference is acknowledged.

Although the animal model paradigm is holding on then, it does not appear to be in good health. Another philosopher of science, Imre Lakatos, observed that a research programme in decline is unable to increase its predictive power or successfully extend itself to new cases; its only changes relate to existing problems and so it falls behind as it attempts to deal with anomalies [23]. It could be argued that all these features are true of the animal research paradigm. Nevertheless, Kuhn argued that even if a paradigm has gone badly astray, it will only be declared invalid if an alternate paradigm is available to take its place. Certainly, NAMs claim to be able to replace animal models. According to Kuhn, if they are to be successful, NAMs will need to solve one or more of the problems that prompted the crisis in the old paradigm—in this case lack of predictivity to humans. Kuhn also suggests that “particularly persuasive arguments can be developed if the new paradigm permits the prediction of phenomena that had been entirely unsuspected while the old one prevailed.” (p153) Having said that, however, he observes that the process of paradigm change is rarely accomplished on the basis of rational argument or evidence alone. Speaking of scientists from different paradigms, he writes: “Though each may hope to convert the other to his way of seeing his science and its problems, neither may hope to prove his case. The competition between paradigms is not the sort of battle that can be resolved by proofs.’ (p. 147) This is partly because, using different language, definitions and standards and often disagreeing about which issues need to be resolved, proponents of competing paradigms find it difficult to communicate with each other. As Kuhn put it, they “practice their trades in different worlds”. (p. 149) Rather than being persuaded by the objective benefits of an alternative paradigm, scientists have to undergo something akin to a “conversion”, suggests Kuhn. He notes that such conversions cannot be forced and that what usually happens is a gradual shift in professional allegiance, until only a few “elderly hold-outs” remain.

Kuhn did not believe that scientists should drop a paradigm every time problems arise; he recognised that ideas need time and protection to develop, and that too much willingness to revise basic beliefs could make science chaotic. Nevertheless, he felt it reasonable to ask how long a research programme should be given to prove itself. Lakatos suggests it may be acceptable to protect a struggling research programme for a while, in case it recovers, but cautions that this is a high-risk strategy [23]. Where there is doubt, suggests Larry Laudan (another philosopher of science), it is rational to pursue the research tradition that has the highest level of problem-solving power [24]. In the context of preclinical research, this would be the tradition with the highest likelihood of benefiting humans. The question of which tradition this might be is, of course, highly contested. Those in favour of animal research point to the ability of animals to provide an intact in vivo system for developing and testing drugs, relating examples of the ways in which animals have benefited humans in the past. Those in favour of NAMs highlight the limitations of animal models and point to the predictive power of human relevant methodologies. Animal models carry the weight of tradition, custom and practice. NAMs promise the future and the opportunity to transform drug discovery. In addition to problem-solving power, however, there are further criteria that might be considered when choosing between competing paradigms.

First, it might be worth reflecting on which paradigm has the ethical advantage. Experiments on animals have always been challenged on ethical grounds; indeed, while the French physiologist Claude Bernard was busy establishing vivisection as part of the scientific method in the 19th century, his wife Marie-Francoise was working alongside a newly established animal protection society to oppose the practice [25]. A proportion of the public has always opposed animal research on ethical ground and this proportion has recently increased; in the US, 52% of the public now opposes the use of animals in scientific research [26], in EU member states 66% of adults agree that the EU should immediately end all animal testing [27], and in the UK 38% of the public believe that animals should not be used in any scientific research because of the importance they place on animal welfare [28]. There is also growing recognition of animal sentience, both generally and within the law [29]. The use of NAMs also raises ethical issues, although these appear to be fewer and relatively easy to resolve, for example there may be ethical issues around tissue donation and consent for use in organ-on-chip models or around the use of patients’ cells for personalised medicines.

Further criteria for choosing between paradigms might include the speed each takes to achieve its goal, which in this case is clinical translation. In the case of animal research, if the quality of animal studies has first to be improved, a delay of many decades seems likely. NAMs also have to be robust of course, but their advantage lies in their direct relevance to humans, meaning that no uncertainty or delay is introduced due to species differences. NAMs could therefore be put to the test much more quickly. Nevertheless, there are still many obstacles to their adoption, including regulatory hurdles, lack of familiarity and a dearth of funding—all of which create delay. Financial cost is another criterion. Under the present system, the average cost of developing a successful new drug is estimated to be $2.6 billion [30], with each new drug taking up to 10 years to develop [31]. NAMs, on the other hand, appear to have potential to reduce costs. It has been estimated, for example, that organ-on-a-chip technologies alone could save between 10–26% in research and development costs per drug [32]. 

In some countries, the animal research paradigm appears to be going strong. In others, there are signs of movement towards NAMs. Here in the UK, the government’s innovation agency has stated that animal studies could be replaced by NAMs for drug efficacy and safety testing [33], while the UK’s Medicines Discovery Catapult has called for the process of drug discovery to be “retooled’ with non-animal, human-relevant technologies [34]. Elsewhere, the Dutch government aims to be a frontrunner in NAMs and has set a 2025 deadline for phasing out the use of animals in the regulatory safety testing of medicines and chemicals [35]. In the US millions of dollars are being invested in NAMs, with the US Food and Drug Administration and the US Environmental Protection Agency (EPA) working together to support and progress NAMs at the highest levels. The US is hosting a world summit on organs-on-a-chip in 2021 and the EPA has recently committed to reducing by 30% the use of mammals in chemical testing by 2025 and to ending their use by 2035, as well as promoting and developing NAMs [36]. The saying goes that when the US sneezes, the rest of the world catches a cold. Whether this happens in relation to NAMs, only time will tell.
